# *Henneguya* (Cnidaria: Myxosporea: Myxobolidae) infections of cultured barramundi, *Lates calcarifer* (Perciformes: Latidae) in an estuarine wetlands system of Malaysia: description of *Henneguya setiuensis* n. sp., *Henneguya voronini* n. sp. and *Henneguya calcarifer* n. sp.

**DOI:** 10.1007/s00436-019-06541-1

**Published:** 2019-11-25

**Authors:** Muhammad Hafiz Borkhanuddin, Gábor Cech, Kálmán Molnár, Faizah Shaharom-Harrison, Tran Nguyen Duy Khoa, Muhammad Arif Samshuri, Suhairi Mazelan, Stephen D. Atkinson, Csaba Székely

**Affiliations:** 1 Faculty of Science & Marine Environment, Universiti Malaysia Terengganu, 21030 Kuala Nerus, Malaysia; 2 Institute for Veterinary Medical Research, Centre for Agricultural Research, Hungarian Academy of Sciences, POB 18, Budapest, H-1581 Hungary; 3 Faculty of Fisheries & Food Science, Universiti Malaysia Terengganu, 21030 Kuala Nerus, Malaysia; 4 Institute of Tropical Aquaculture & Fisheries Research (AKUATROP), Universiti Malaysia Terengganu, 21030 Kuala Nerus, Malaysia; 5 Department of Coastal Aquaculture, Can Tho University, Ninh Kieu District, Can Tho City, Viet Nam; 6 Department of Microbiology, Oregon State University, Corvallis, Oregon, 97330 USA

**Keywords:** Cnidaria, *Henneguya*, Setiu Wetlands, Barramundi, South China Sea

## Abstract

Examination of 35 barramundi (*Lates calcarifer*) from aquaculture cages in Setiu Wetland, Malaysia, revealed a single fish infected with three *Henneguya* spp. (Cnidaria: Myxosporea). Characterization of the infections using tissue tropism, myxospore morphology and morphometry and 18S rDNA sequencing supported description of three new species: *Henneguya setiuensis* n. sp., *Henneguya voronini* n. sp. and *H. calcarifer* n. sp*.* Myxospores of all three species had typical *Henneguya* morphology, with two polar capsules in the plane of the suture, an oval spore body, smooth valve cell surfaces, and two caudal appendages. Spores were morphometrically similar, and many dimensions overlapped, but *H. voronini* n. sp. had shorter caudal appendages compared with *H. calcarifer* n. sp. and *H. setiuensis* n. sp. Gross tissue tropism distinguished the muscle parasite *H. calcarifer* n. sp. from gill parasites *H. setiuensis* n. sp. and *H. voronini* n. sp.; and these latter two species were further separable by fine-scale location of developing plasmodia, which were intra-lamellar for *H. setiuensis* n. sp. and basal to the filaments for *H. voronini* n. sp. small subunit ribosomal DNA sequences distinguished all three species: the two gill species *H. setiuensis* n. sp. and *H voronini* n. sp. were only 88% similar (over 1708 bp), whereas the muscle species *H. calcarifer* n. sp. was most similar to *H. voronini* n. sp. (98% over 1696 bp). None of the three novel species was more than 90% similar to any known myxosporean sequence in GenBank. Low infection prevalence of these myxosporeans and lack of obvious tissue pathology from developing plasmodia suggested none of these parasites are currently a problem for barramundi culture in Setiu Wetland; however additional surveys of fish, particularly at different times of the year, would be informative for better risk assessment.

## Introduction

Myxosporeans (Cnidaria: Myxosporea) are common, diverse parasites of marine and freshwater fishes (Lom and Dyková 2006). The genus *Henneguya* Thélohan, 1892, in family Myxobolidae, is the third most speciose myxosporean genus after *Myxidium* and *Myxobolus*, with > 200 *Henneguya* species described (Eiras 2002; Eiras and Adriano 2012; Székely et al. 2018). While most *Henneguya* species do not cause overt disease in their hosts, a few are economically important pathogens. Infections can cause mortalities in fish populations if parasites replicate to high intensities on the gills and cause respiratory insufficiency, especially in juvenile fish (Haaparanta et al. 1994; Whitaker et al. 2005). For example, *H. ictaluri* Pote, Hanson et Shivaji, 2000, causes proliferative gill disease in cultured channel catfish (*Ictalurus punctatus* Rafinesque) with up to 50% mortality (Bowser and Conroy 1985; Bosworth et al. 2003), and *H. lateolabracis* Yokoyama, Kawakami, Yasuda et Tanaka, 2003, is the causative agent of fatal cardiac henneguyosis in cultured Japanese seabass (*Lateolabrax* spp.) (Pote et al. 2000; Yokoyama et al. 2003). *Henneguya* infection in the muscle can reduce the quality and marketability of the fish (Patashnik and Groninger 1964; Boyce et al. 1985), while other *Henneguya* species, for example, *H. testicularis* and *H. oviperda*, which are commonly found in the gonads of farmed fish, may affect reproductive health (Sitjá-Bobadilla 2009; Sokolov et al. 2019). *Henneguy*a prevalence of infection can reach 75–100%, with an intensity of up to 8,000 cysts in one fish (Nie 1996; Fomena and Bouix 1996). Whether intense or not, exposure of the fish host to other stressors can exacerbate disease effects of the parasite, for example, under stressful or aggregated conditions, mortality of 95–100% has been reported in farm-reared channel catfish (Bowser and Conroy 1985; Minchew 1972).

Previous studies have demonstrated that *Henneguya* spp. are the most common myxosporean parasites in non-cyprinid fishes in Southeast Asia (Molnár et al. 2006a, 2006b; Székely et al. 2009a, 2009b). From Malaysia, the few known *Henneguya* species are from freshwater host species and/or habitats (Shariff 1982; Molnár et al. 2006a, 2006b; Székely et al. 2009a, 2009b). Six species are described from non-cyprinid fishes in Malaysia: *H. shariffi* Molnár, Székely, Mohamed and Shaharom-Harrison, 2006, in *Pangasianodon hyphopthalmus* Sauvage (Pangasidae: Siluriformes); *H. mystusia* Sarkar, 1985, in *Hemibagrus nemurus* Valenciennes (Bagridae: Siluriformes); *H. basifilamentalis* Molnár, Székely, Mohamed and Shaharom-Harrison, 2006, in *H. nemurus*; *H. shaharini* Shariff 1982, in *Oxyeleotris marmorata* (Bleeker) (Eleotridae: Perciformes); and *H. hemibagri* Tchang et Ma, 1993, in *H. nemurus*, all from the municipality of Kuala Terengganu, and *H. daoudi* Székely, Shaharom-Harrison, Mohamed and Molnár, 2009 in *Trichopodus trichopterus* (Pallas) (Osphronemidae: Perciformes), from Machang municipality. This research on the biodiversity of *Henneguya* spp. in freshwater habitats contrasts sharply with the paucity of information on these parasites from marine or estuarine fishes (Eiras 2002; Dyková et al. 2011).

Fishes of the genus *Lates* include species that are economically important such as *Lates calcarifer* (Barramundi) or *Lates niloticus* (Nile perch), which have been cultured globally as popular food fish (Tantikitti et al. 2005; Biswas et al. 2010; Aloo et al. 2017). Barramundi is cultured primarily in sea cages near river mouths or estuaries and harvested throughout the year in the Indo-pacific region (Boonyaratpalin 1997; Tantikitti et al. 2005; Biswas et al. 2010). Despite their importance, very little myxosporean research has been conducted on these fishes. Only one *Henneguya* species is known from barramundi, *H. latesi* Tripathi 1952, from India, together with several other myxosporeans: *Myxidium calcariferi* Chakravarty, 1943, *Myxidium procerum* var. *calcariferi* Chakravarty, 1943, and *Myxobolus calcariferum* Basu and Haldar, 2003 (Tripathi 1952; Eiras et al. 2005; Eiras et al. 2011).

*Henneguya* has never been documented formally in cultured barramundi in Setiu Wetland, Terengganu, Malaysia, despite recurring observations (2009–2012) of spores in gills, but never from the muscle (Shaharom-Harrison pers. comm.). These observations prompted us to formally identify the species and determine if the parasite/s were causing any pathology in the barramundi, which are cultured at high densities with other important fish species like groupers (*Epinephelus* sp.). We conducted a limited survey of myxosporean parasites in cultured barramundi and identified three new *Henneguya* species: *H. setiuensis* n. sp. and *H. voronini* n. sp. from different locations in the gills and *H. calcarifer* n. sp. from the skeletal muscle.

## Materials and methods

### Host and parasite samples

Cultured barramundi, *Lates calcarifer* (n = 35; length 15–30 cm), were sampled from estuarine net cages from Setiu Wetland, Malaysia (5^0^40’53.30”N, 102^0^ 42’43.93”E). Specimens were transported live to the Institute of Tropical Aquaculture (AKUATROP), University Malaysia Terengganu (UMT), in March 2013, and maintained in an aerated fish tank. Within 5 days of collection, fish were sedated using clove oil and killed by a cervical cut, according to institutional animal treatment protocols. Fish were necropsied and surveyed for myxosporean parasites with a Leica EZ4 stereomicroscope and a Leica DM750 compound microscope. We examined the body surface, oral cavity, gills, heart, gall bladder, kidney and skeletal muscle for overt myxosporean pseudocysts (plasmodia). When suspected myxosporean plasmodia were found, they were excised using fine forceps and then ruptured in a drop of phosphate-buffered saline (PBS) and the contents examined in wet mount preparations. Parasites were examined and photographed using a Nikon Model Eclipse 80i advanced light microscope. Spores were checked for the presence of an iodinophilous vacuole after adding a drop of Lugol’s iodine solution. At least 2 dimensions (length, width or thickness) were measured from 30 or more spores from each plasmodium, according to the guidelines of Lom and Arthur (1989), with the exception that we use the more structurally accurate term “polar tubule” instead of “polar filament”. Spores from individual plasmodia were preserved in 80% ethanol for subsequent molecular analysis. For histological examination, tissue samples from infected organs containing mature plasmodia were fixed in Bouin’s solution, embedded in paraffin wax, sectioned at 5–6 μm and stained with haematoxylin and eosin.

### Molecular and phylogenetic analyses

At the IVMR CAR HAS, Hungary, DNA was extracted from the ethanol fixed spores using a DNeasy tissue kit (animal tissue protocol; Qiagen, Germany) according to the manufacturer’s instructions. Two rounds of PCR were performed: round 1 with universal eukaryotic small subunit ribosomal DNA (18S rDNA) primers ERIB1 and ERIB10 and fully nested round 2 with primers Myx1F and SphR (Table 1). First-round amplifications were performed in 25 μl reactions that comprised 2 μl extracted DNA ( ~ 0.2 μg), 0.2 mM of each dNTP (MBI Fermentas), 0.5 μM each of the forward and reverse primers, 2.5 μl 10× Taq buffer (MBI Fermentas), 2 U Taq polymerase (MBI Fermentas) and 15 μl of water. Thermal cycling comprised initial denaturation at 95 °C for 3 min, followed by 35 cycles of 1 min at 95 °C, 1 min at 55 °C and 2 min at 72 °C, completed with terminal extension for 7 min at 72 °C and then resting at 4 °C.

**Table 1:** Primers used for PCR and sequencing

Primer	Sequence	Application	Reference
ERIB1	5’-ACCTGGTTGATCCTGCCAG-3’	1st round PCR	Barta (1997)
ERIB10	5’-CTTCCGCAGGTTCACCTACGG-3’	1st round PCR	Barta (1997)
Myx1F	5′-GTG AGA CTG CGG ACG GCT CAG-3′	2nd round PCR	Hallett and Diamant (2001)
SphR	5′-GTT ACC ATT GTA GCG CGC GT-3′	2nd round PCR and sequencing	Eszterbauer and Székely (2004)
MC5	5′-CCT GAG AAA CGG CTA CCA CAT CCA-3′	Sequencing	Molnár et al. (2002)
MC3	5′-GAT TAG CCT GAC AGA TCA CTC CAC A-3′	Sequencing	Molnár et al. (2002)
HMF1	5′-GAT CTG GTG ATG AGT GGT GCA T-3′	Sequencing	Present study
HMF2	5′-GAG TTG TTC AAT GCT CGG GAT-3′	Sequencing	Present study
HMR1	5′-GGC CAT CCT TAC GCG CAA TTA -3′	Sequencing	Present study
HMR2	5′-GCA ACG TCG AAC CAA AGC GAT-3′	Sequencing	Present study

Second-round PCR was conducted in 50 μl, which consisted of 1 μl amplified DNA ( ~ 0.5 μg), 0.2 mM of each dNTP (MBI Fermentas), 0.5 μM each of the forward and reverse primers, 5 μl 10 × Taq buffer (MBI Fermentas), 2 U Taq polymerase (MBI Fermentas) and 33 μl of water. Amplification conditions in the second round were 95 °C for 3 min, followed by 35 cycles of 95 °C for 50 s, 50 °C for 50 s and 72 °C for 1 min 40 s, with terminal extension of 72 °C for 10 min, and then rest at 4 °C. All PCRs were performed in a PTC-200 thermocycler (MJ Research).

Amplified DNA was purified with EZ-10 Spin Column PCR Purification Kit (Bio Basic Inc., USA) and then sequenced in both directions using an ABI BigDye Terminator v3.1 Cycle Sequencing Kit with an ABI 3100 Genetic Analyser (IVMR, HAS). Initial short ( ~ 800 bp) sequences inspired development of new *Henneguya-*specific sequencing primers HMF1, HMF2, HMR1 and HMR2 (Table 1).

BLAST searches were conducted to determine affinities to other myxosporeans in NCBI GenBank, and we selected the 33 18S rDNA sequences that had above 80% similarity to our three novel *Henneguya* species. These sequences were aligned using Clustal W (Thompson et al. 1994). Alignments were corrected manually in MEGA X (Kumar et al. 2018). The dataset was tested using MEGA X for the nucleotide substitution model of best fit as indicated by the Akaike Information Criterion (AIC). Phylogenetic relationships were inferred using the Maximum Likelihood (ML) method with the GTR + G + I substitution model and bootstrapping with 1000 replicates. *Chloromyxum cyprini* (AY604198) was chosen as the outgroup, as this represents one of the most primitive lineages of the myxosporean group that includes *Henneguya* spp. Genetic distances were determined using the *p*-distance model matrix in MEGA X.

### Scanning electron microscopic (SEM)

Plasmodia of *Henneguya voronini* n. sp. were compressed and ruptured onto 25 × 40 mm glass slides, fixed in 2.5% glutaraldehyde, then washed several times in 0.1 M sodium cacodylate buffer and postfixed in cold 1% (w/v) osmium tetroxide. Fixed tissue was then dehydrated in an alcohol series of 35% to 100% for 10- to 15-min each step, and then critical point dried in carbon dioxide (Baltac CPD 030). The glass slide containing the dried specimens was cut into smaller pieces, mounted onto aluminium stubs with double-sided tape and then sputter-coated with gold (Jeol JFC 1600). The specimens were examined in a Jeol SEM (Model JSM 6360LA).

## Results

Thirty-five barramundi were examined, and myxosporean infections were found in only a single fish. Plasmodia were found in two different organs, the gills and the muscle. Plasmodia in the gills occurred in two well-differentiated locations: spherical plasmodia 50–70 μm in diameter developed intra-lamellarly, whereas ellipsoidal plasmodia 250–300 × 130–150 μm were located basi-filamentally between gill filaments. A third type of plasmodium, which was ovoid and 300–400 μm long, was found within the skeletal muscle. All three types of plasmodia contained mature myxospores, which were morphologically similar, with compact, ellipsoidal spore bodies of typical *Henneguya* morphology, with two polar capsules in the plane of the suture, smooth surfaced valve cells, with caudal appendages that extended about four times longer than the spore body. Morphometrics (Table 2) overlapped among myxospores from the three different tissue locations. Our diagnosis of three different species was based on morphological comparisons with existing records, specific tissue site of development and 18S rDNA sequence data (see below for taxonomic summaries).

**Table 2:** Comparison of hosts, localities, tissues and myxospore dimensions of *Henneguya* species described from *Lates* sp. *SL* spore length, *SW* spore width, *AL* caudal appendage length, *ST* spore thickness, *PCL* polar capsule length, *PCW* polar capsule width, *NC* number of coils of unfired polar tubule. All measurements are in μm

Species	SL	SW	ST	AL	PCL	PCW	NC	Plasmodium dimension	Plasmodium shape	Site of infection	Host	Locality	References
*H. mandouri*	11–13*	6.0–7.5	–	40–53	3.1–4.3	1.5–2.2	3–5	200–1000 × 100–500	Ellipsoidal or oval	Gill filaments	*L. niloticus*	Egypt	Rabie et al. 2009
*H. massii*	8–9 (8.3 ± 0.1) **	5–6 (5.6 ± 0.1)	–	12–14 (13.6 ± 0.3)	2–3 (2.8 ± 0.1)	1–2 (1.6 ± 0.1)	–	–	Spherical	Gill filaments	*L. niloticus*	Chad	Kostoïngue et al. 2001
*H. ghaffari*	11.8–14 (13.0 ± 0.6)	6.9–7.9 (7.5 ± 0.4)	4.9–5.9 (5.2 ± 0.5)	36.3–53.0 (43.2)	4.8–5.9 (5.2 ± 0.5)	2.8–3.9 (3.2 ± 0.3)	4–5	1000 × 400	Spindle shaped in the gill; round to oval in the intestine	Gill filaments, intestinal muscles	*L. niloticus*	Egypt	Ali 1999
*H. mbakaouensis*	10–12 (10.8)	7–9.9 (7.5)	4.8–5.2 (5.0)	51.5–69.2 (61.8)	3.5–4.7 (4.0)	2–3 (2.5)	4–5	120–470 × 60–230	Ovoid or subspherical	gills	*L. niloticus*	Cameroon	Fomena and Bouix 2000
*H. latesi*	9.0–10.8	6.3–8.2	5.4	17.2–25.4	3.6	2	–	–	–	Gills, mouth	*L. calcarifer*	India	Tripathi 1952
*H. setiuensis* n. sp.	8.3–9.5 (8.9 ± 0.4)*	5.8–6.0 (5.9 ± 0.3)	4.1–4.2 (4.1 ± 0.1)	28.0–32.0 (30.5 ± 1.6)	3.1–3.5 (3.3 ± 0.2)	2.0–2.2 (2.1 ± 0.1	6	50–75 × 50–75	Spherical	Between the gill lamellae	*L. calcarifer*	Malaysia	Present study
*H. voronini* n. sp.	9.5–10.3 (9.9 ± 0.3)*	6.3–7.3 (6.8 ± 0.3)	–	25.0–29.0 (27.2 ± 1.4)	3.5–4.0 (3.7 ± 0.2)	2.0–2.2 (2.1 ± 0.1)	6	250–300 × 130–150	Ellipsoidal	At the base of gill filaments	*L. calcarifer*	Malaysia	Present study
*H. calcarifer* n. sp.	8.3–10.0 (9.4 ± 0.6)*	4.8–5.5 (5.2 ± 0.3)	3.7–4.0 (3.8 ± 0.1	28.0–35.0 (30.9 ± 3.0)	3.1–3.7 (3.4 ± 0.2)	1.1–1.7 (1.4 ± 0.2)	6	300 × 400	Spherical	Skeletal muscles	*L. calcarifer*	Malaysia	Present study

*oval in frontal view with rounded anterior end

**oval in frontal view with rounded anterior and posterior ends

### *Henneguya setiuensis* n. sp.

Type host: barramundi, *Lates calcarifer* (Bloch 1790).

Site of infection: Within gill lamellae.

Locality: Setiu Wetlands, Terengganu, Malaysia.

Prevalence of infection: 2.8% (1/35).

Vouchers: Digital images of syntype spores and histological sections deposited in the parasitological collection of the Zoological Department, Hungarian Natural History Museum, Budapest, collection no. HNHM-71892. The 18S rDNA sequence was deposited in GenBank under accession number MH743111.

Etymology: The species is named after the collection location, Setiu Wetlands, Malaysia.

**Fig. 1:**
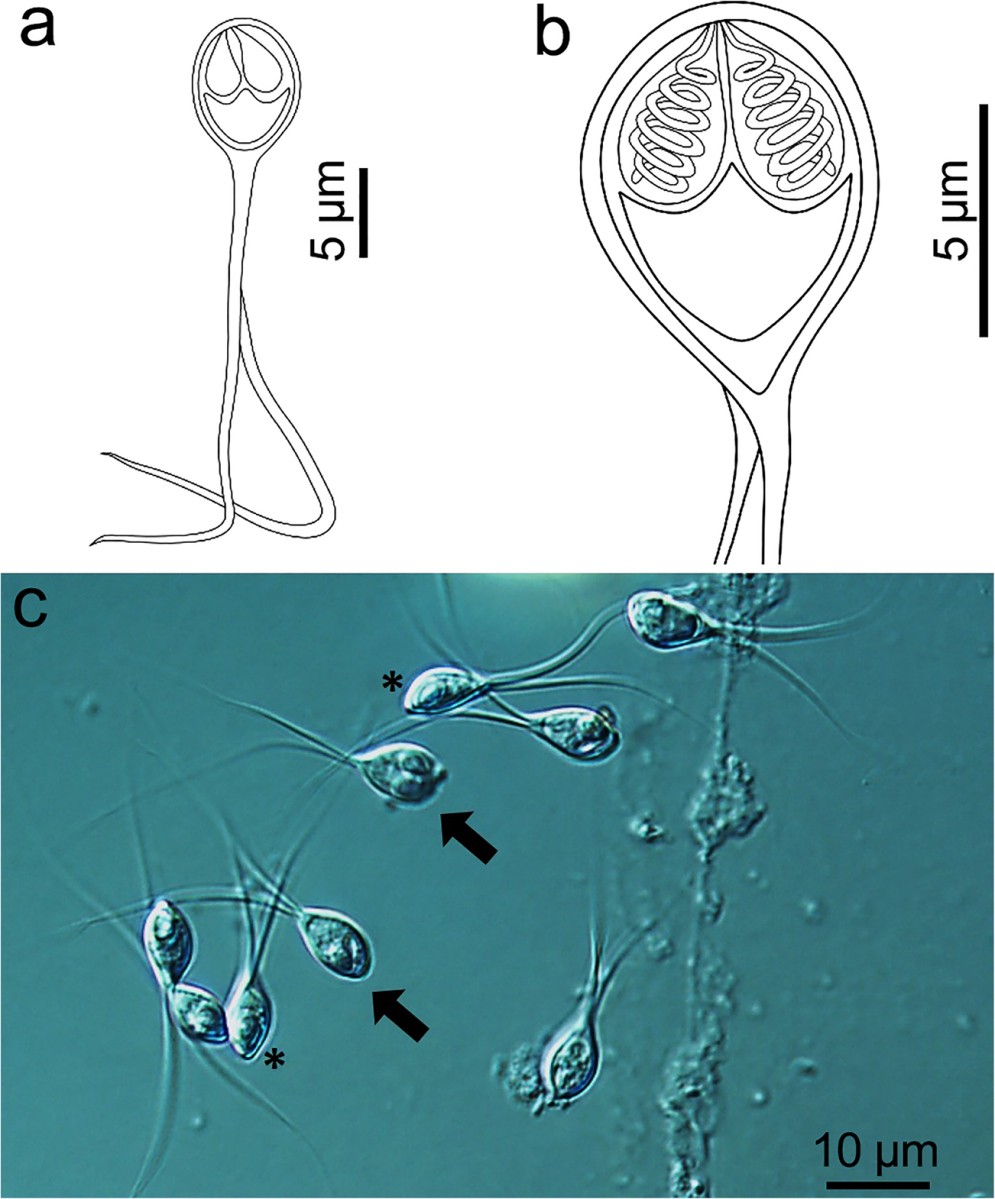
Henneguya setiuensis n. sp. (**a**–**b**) Line drawings of mature myxospores in frontal view showing polar capsules with coiled polar tubules. (**c**) Fresh, unstained myxospores in frontal (arrow) and sutural (*) views, with divergent caudal appendages

Description of spores: Spores symmetric, with two equal caudal appendages, fusiform sporebody, two equal-sized pyriform polar capsules (Fig. 1). Spore wall thin (0.3–0.4 μm), smooth and composed of two equal valves. Apical end of spore body blunt, caudal end tapered and extends into the caudal appendages. Fresh myxospores 36–41 μm long, spore length 8.9 ± 0.4 (8.3–9.5) μm, width 5.9 ± 0.3 (5.8–6.0) μm and thickness 4.1 ± 0.1 (4.1–4.2) μm. Two polar capsules, pyriform, blunt at the posterior end and taper anteriorly, length 3.3 ± 0.2 (3.1–3.5) μm and width 2.1 ± 0.1 (2.0–2.2) μm. Polar tubules coiled in 6 turns perpendicular to the long axis of the polar capsules. Sporoplasm binucleate with a small iodinophilous vacuole. Caudal appendages straight, tapering, 30.5 ± 1.6 (28.0–32.0) μm. Plasmodia spherical, diameter 50–75 μm.

Remarks: We identified *H. setiuensis* n. sp. as a new species based on host, site of development (intra-lamellar in gills), morphology and morphometrics. The spores were most similar morphometrically and by tissue tropism (gill) to a previously described species *H. latesi* Tripathi 1952, from barramundi in India; however, the description of this earlier species has limited informative characters for comparison of spore measurements, and no 18S rDNA sequence data (Table 2). From the limited data available for comparison, *H. setiuensis* n. sp. was distinct in spore body shape (rounder vs oval), total length (36–41 μm vs 26–36 μm) and spore thickness (4.1 μm vs 5.4 μm). Determination of whether *H. latesi* represents a taxon distinct from any that we observed in Malaysian barramundi would require rediscovery of parasite material from the type locality in India, with molecular and fine-scale tissue tropism observations. Compared with *Henneguya* spp. known from *Lates* species hosts, *H. setiuensis* n. sp. was unique in most dimensions (Table 2) and had the smallest plasmodia; however molecular data and specific tissue site of infection are not available for these other species for more precise comparison. *H. setiuensis* n. sp. was morphometrically similar to the other two species found in the same fish in the current study, except spore width, polar capsule width and plasmodium size, but distinguishable based on fine-scale tissue tropism and DNA sequence.

Molecular analysis: Our consensus 18S rDNA sequence of 1708 bp was shown by BLAST search to be most similar to other *Henneguya* species in GenBank, but all < 89%. Pairwise comparison showed *H. setiuensis* n. sp. to be no more than 91% similar to the other *Henneguya* species found in the same fish: 89.9%, *p*-distance 0.101 with *H. calcarifer* n. sp.; 90.1%, *p*-distance 0.099 with *H. voronini* n. sp.

Histology: Plasmodia observed in different parts of the gill filaments, inside the multilayered epithelium between lamellae (Fig. 4a–b ). Only local damage of the lamellae was observed, with no gross pathology of the gill filaments.

### *Henneguya voronini* n. sp.

Type host: barramundi, *Lates calcarifer* (Bloch 1790).

Site of infection: Base of the gill filament.

Type locality: Setiu Wetlands, Terengganu, Malaysia.

Prevalence of infection: 2.8% (1/35).

Type material: Digital images of syntype spores and histological sections were deposited in the parasitological collection of the Zoological Department, Hungarian Natural History Museum, Budapest, collection no. HNHM-71893. The 18S rDNA sequence was deposited in GenBank under accession number MH743110.

Etymology: The species is named in honour of V. A. Voronin, an eminent Russian fish parasitologist.

**Fig. 2:**
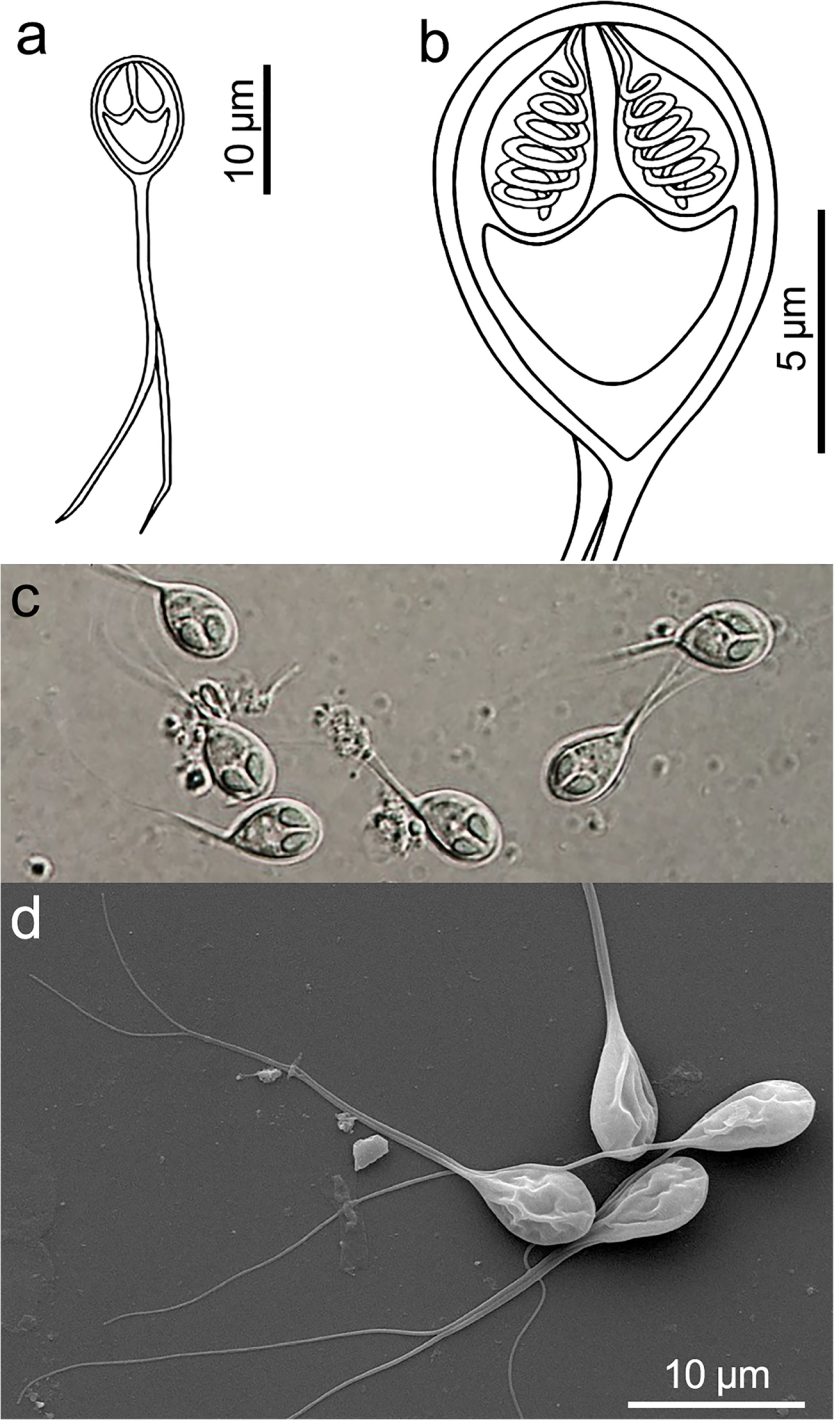
Henneguya voronini n. sp. (**a**–**b**) Line drawings of mature myxospores in frontal view showing polar capsules with coiled polar tubules. (**c**) Fresh, unstained myxospores in frontal view showing the two pyriform polar capsules. (**d**) Scanning electron microscope image of the spores showing simple, smooth valve cell surfaces, each contiguous with a caudal process; features typical of the genus

Description of spores: Myxospores symmetric, with two equal caudal appendages and equal-sized polar capsules (Fig. 2). Spore wall thin (0.3–0.4 μm), smooth and composed of 2 equal valves. Apical end of spore body blunt, the caudal end tapers and extends into the caudal appendages. Total length 35–39 μm, length of spore 9.9 ± 0.3 (9.5–10.3) μm and width 5.9 ± 0.3 (5.8–6.0) μm; thickness could not be measured as no spores were observed in sutural plane. Polar capsules pear shaped, blunt at the posterior end and tapered anteriorly, length 3.7 ± 0.2 (3.5–4.0) μm and width 2.1 ± 0.1 (2.0–2.2) μm. Polar tubules coiled in 6 turns perpendicular to the long axis of the polar capsules. Sporoplasm binucleate, with a small iodinophilous vacuole. Caudal appendages straight, tapering, length 27.2 ± 1.4 (25.0–29.0) μm, about 4 times as long as the spore body. Plasmodium ellipsoidal 250–300 μm × 130–150 μm.

Remarks: Myxospores of *H. voronini* n. sp. could not be distinguished morphometrically using most measurements with the two other species observed in the host. Both *H. voronini* n. sp. and *H. setiuensis* n. sp. were found in the gills but the specific tissue locality differed: the large ellipsoidal plasmodia (up to 300 μm) of *H. voronini* n. sp. were localized to the cartilaginous base of the gill filaments, while the smaller spherical plasmodia of *H. setiuensis* n. sp. (up to 75 μm) developed between lamellae of the gill filaments. Considering *Henneguya* spp. from other *Lates* species, spores of *H. voronini* n. sp. could be differentiated in at least two dimensions (Table 2).

Molecular analysis: 1696 bp 18S rDNA were sequenced, including the primers. A BLAST search indicated that highest sequence similarities were to other *Henneguya* species in GenBank, but all < 90%. Pairwise analysis showed *H. voronini* n. sp. was molecularly very similar to *H. calcarifer* n. sp. (97.7%; 1658/1969 bp; *p*-distance 0.013), described from the same fish (below).

Histology: Ellipsoidal plasmodia were located in the cartilaginous gill arch between gill filaments (Fig. 4c). We suspect that development began in the multilayered connective tissue covering the gill arch and then a large part of the plasmodium moved into the gill filaments and was covered by a multilayered epithelium as it matured.

Microscopy: SEM revealed that the valve cell surfaces were smooth, which is a morphological feature typical of genus *Henneguya*.

### *Henneguya calcarifer* n. sp.

Type host: barramundi, *Lates calcarifer* (Bloch 1790).

Site of infection: Skeletal muscle.

Type locality: Setiu Wetlands, Terengganu, Malaysia.

Prevalence of infection: 2.8% (1/35).

Type material: Digital images of syntype spores were deposited in the parasitological collection of the Zoological Department, Hungarian Natural History Museum, Budapest, collection no. HNHM-71894. The 18S rDNA sequence was deposited in GenBank under accession number MH743109

Etymology: The species is named after the host.

**Fig. 3:**
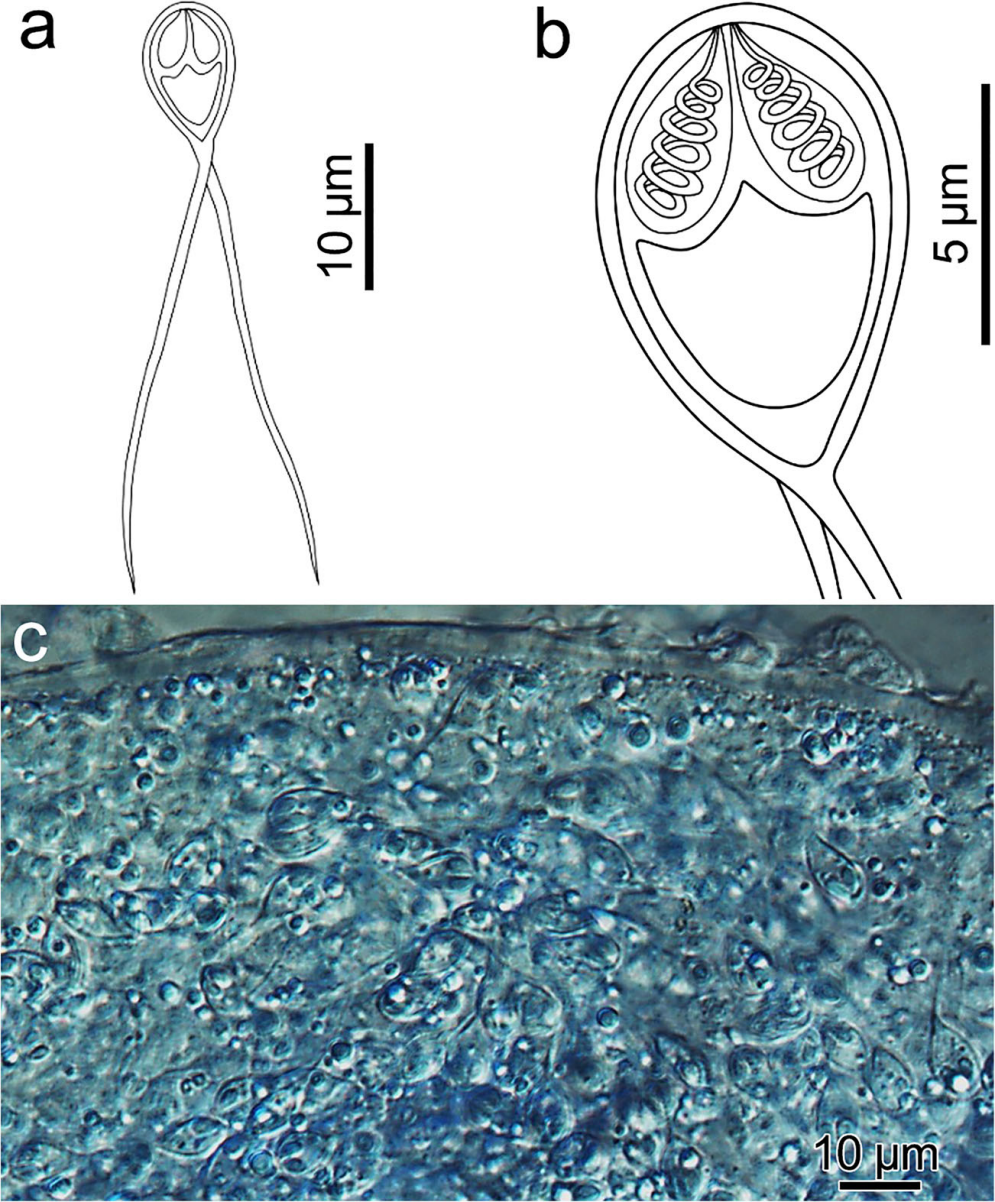
*Henneguya calcarifer* n. sp. (**a**–**b**) Line drawings of mature myxospores in frontal view showing polar capsules with coiled polar tubules. (**c**) Unstained, compressed plasmodium, densely packed with mature myxospores

Description of spores: Fig. 3. Myxospores symmetric, with two equal caudal appendages, and equal-sized polar capsules. Spore wall 0.3–0.4 μm, smooth and composed of two equal valves. Apical end of spore body blunt, the caudal end tapers and extends into the caudal appendages, total length 34–45 μm. Spore length 9.4 ± 0.6 (8.3–10.0) μm, width 5.2 ± 0.3 (4.8–5.5) μm and thickness 3.8 ± 0.1 (3.7–4.0) μm. Two polar capsules pear shaped, blunt at the posterior end and taper anteriorly, length 3.4 ± 0.2 (3.1–3.7) μm and width 1.4 ± 0.2 (1.1–1.7) μm. Polar tubules coiled in 6 turns perpendicular to the long axis of the capsule. Sporoplasm binucleate with a small iodinophilous vacuole. Caudal appendages straight, tapering, length 30.9 ± 3.0 (28.0–35.0) μm, ~ 4 times longer than the spore body. Plasmodia spherical 300 × 400 μm.

**Fig. 4:**
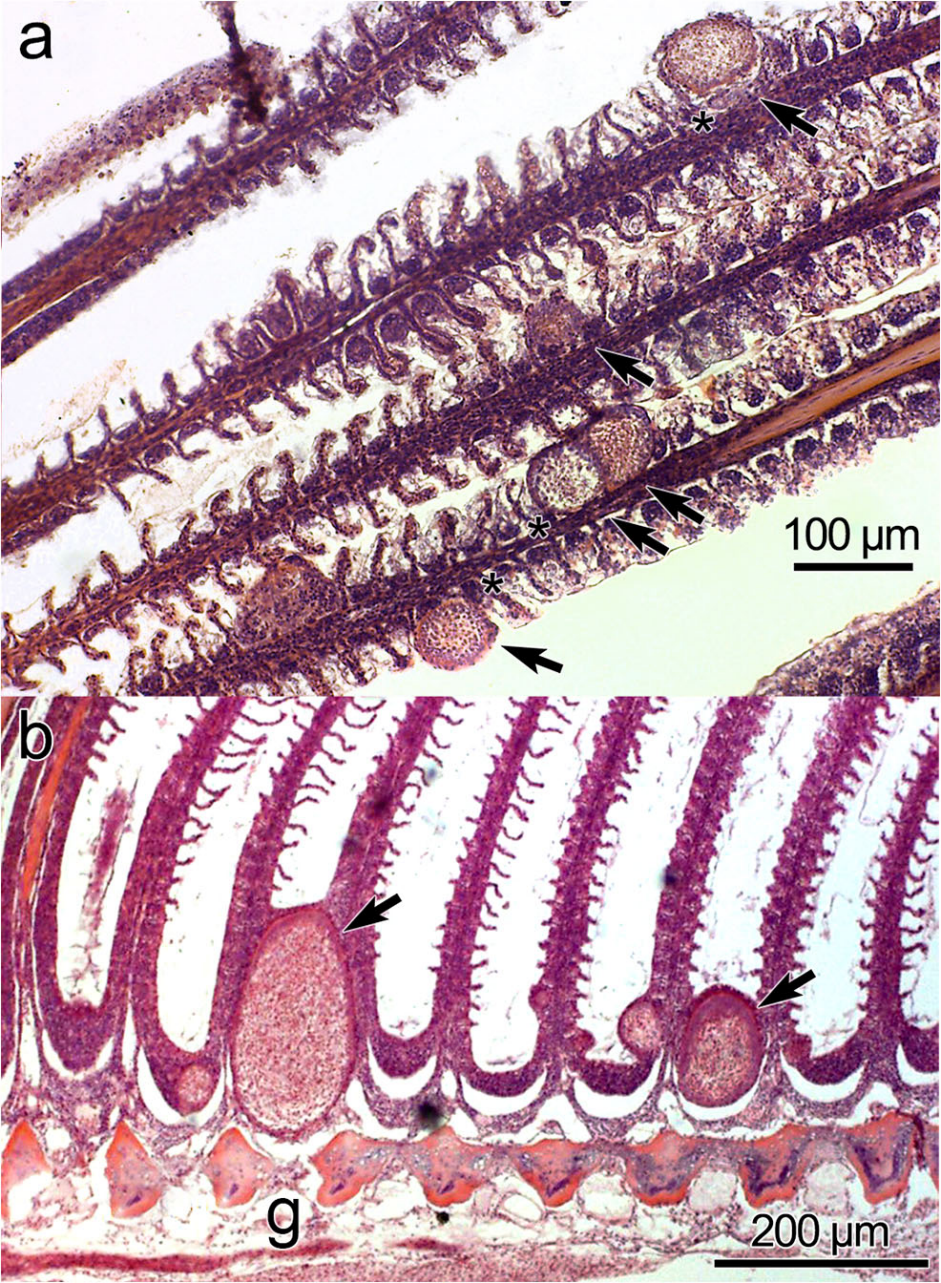
Histological sections of gill filaments from *Lates calcarifer* infected by plasmodia of *H. setiuensis* n. sp. and *H. voronini* n. sp. (**a**) Plasmodia of H. setiuensis n. sp. (arrows) producing compression and damage to the lamellae (*). (**b**) Development of plasmodia of *H. voronini* n. sp. (arrows) in the sub-epithelial layer at the base of the filament. Note that the plasmodia also impinge into the gill arch (**g**)

**Fig. 5:**
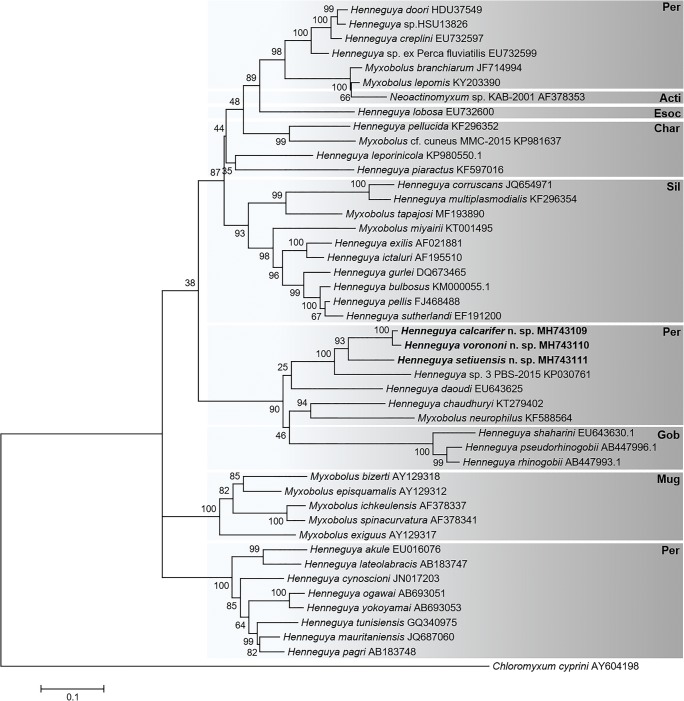
Phylogenetic tree generated by maximum likelihood analysis of 18S ribosomal DNA sequences of Henneguya species from perciform hosts and other closely related myxosporean species identified by BLAST; GenBank accession numbers shown after the species name, including the three novel data in bold (*H. setiuensis* n. sp., *H. voronini* n. sp. and *H. calcarifer* n. sp.). Numbers at nodes indicate the bootstrap confidence values (ML). Taxonomic orders of the fish hosts are shown at right: *Char* Characiformes, *Sil* Siluriformes, *Esoc* Esociformes, *Mug* Mugiliformes, *Gob* Gobiiformes, *Per* Perciformes and *Acti* Actinospores. *Chloromyxum cyprini* was used as an outgroup

Remarks: *H. calcarifer* n. sp. resembles morphologically and morphometrically both *H. setiuensis* n. sp. and *H. voronini* n. sp. (Table 2) but has different tissue site of development (muscles not gill). This is the first muscle-infecting *Henneguya* described from *Lates calcarifer* and is relatively distinct from the only other species from a *Lates* congener: *H. ghaffari* from muscle and gills of *L. niloticus* in Egypt (Table 2).

Molecular analysis: 1696 bp 18S rDNA were sequenced. A BLAST search indicated that the most similar species were other *Henneguya* species, but all < 89%. Pairwise analysis showed *H. calcarifer* n. sp. was molecularly very similar (97.7% over 1696 bp) with *H. voronini* n. sp., described from the same fish (above).

Histology: Low intensity of parasite plasmodia in the host skeletal muscle meant no plasmodia were visible in histological sections.

### Phylogenetic analysis

Phylogenetic analyses of 43 myxosporean sequences revealed a topology that showed correlation of myxosporeans clustering with host order, with *Henneguya* spp. that infect Perciformes clustering into three distinct clades. The three species described herein cluster together within a subclade that includes another species that infect Perciformes (Fig. 5).

## Discussion

*Henneguya* is histozoic myxosporean parasite of freshwater and marine fishes and one of the most species-rich genus of myxosporeans with more than 200 species described (Lom and Dyková 2006; Eiras 2002; Eiras and Adriano 2012). We used characters of specific host, tissue, myxospore morphology and 18S rDNA sequences to describe three *Henneguya* species in barramundi, *Lates calcarifer*, from the east coast of Peninsula Malaysia. *Henneguya setiuensis* n. sp. and *H. voronini* n. sp. developed between gill lamellae and at the base of the gill filaments, respectively, while *H. calcarifer* n. sp. developed in skeletal muscle from the flank of the fish. Discovery of the *Henneguya* infections in gills was not unexpected, as this organ is a common site of myxobolid infections; however, *H. calcarifer* n. sp. is the first myxosporean with a preference for muscle in a species of *Lates*. Muscle tropism of *Henneguya* species is less common than gills, being a character of 8 of some 180 species, including only 1 from a host in the Asian region: *H. ophicephali* Chakravarty, 1939, in *Ophiocephalus punctatus* from India (Eiras 2002; Eiras and Adriano 2012).

*Henneguya* has the distinctive morphological character of two caudal appendages, which distinguishes it from the other genera of the family Myxobolidae. However, molecular (SSU rDNA) evidence shows that genus *Henneguya* is polyphyletic, and this does not support the hypothesis that the presence of caudal appendages is a valid character for distinguishing species of *Henneguya* from *Myxobolus* (see comprehensive analysis in Liu et al. 2019). While we acknowledge the weak support for *Henneguya* as a distinct genus, formal taxonomic revision of genera within Myxobolidae is still lacking and is outside the scope of the present study. Accordingly we described the three novel species herein as *Henneguya*, based on the historical morphological character-based definition, augmented with DNA data.

Our PCR approach for genetically characterizing the parasites was based on previous work to amplify DNA from another Malaysian *Henneg*uya species, *H. daoudi*, in which its 18S rDNA was sequenced using “freshwater” myxosporean primers SphF-SphR, MC3-MC5 and MB5r-MB5f (Székely et al. 2009a). Although we were able to amplify ~ 800 bp fragments using SpHR, MC3 and MC5, these did not overlap sufficiently to construct contigs. Accordingly, we used our sequence information from *H. calcarifer* n. sp. to design new barramundi-*Henneguya*-specific primer pairs HMF-HMF2 and HMR1-HMR2, to give sufficient coverage to assemble contigs of 1696–1708 bp, including terminal primers MYX1f and SphR.

*Henneguya* species can cause disease and mortality in farmed fish species, for example, *H. exilis* in catfish (Current and Janovy 1977) and *H. piaractus* in pacu (Martins et al. 1997). Our study of parasites of *L. calcarifer* was to assess the baseline occurrence of myxosporeans and any associated pathology in this important aquaculture species in Malaysia. We observed infection in only 1/35 fish (2.8%), but this individual was positive for all three *Henneguya* species. This was lower than prevalences of other *Henneguya* infections in *Lates* spp.: *H. mandouri* 32/40, 80%; *H. ghaffari* 65/188, 34.6%; *H. latesi* 6/20, 30%; and *H. massii* 3/67 (4.4%) (Tripathi 1952; Ali 1999; Kostoïngue et al. 2001; Rabie et al. 2009). In addition to low prevalence, the single fish that we observed with the infections did not have any observable pathology associated with the parasites. This is consistent with the *Henneguya* infections from congeneric hosts, with none of these reported to cause henneguyosis (Tripathi 1952; Ali 1999; Kostoïngue et al. 2001; Rabie et al. 2009).

Correlation between particular myxosporean species and tissue tropism in the vertebrate host is well-established (e.g. Molnár 1994), particularly in species of Myxobolidae, with fine-scale tissue preferences being observed in the gills (Molnár 2002; Eszterbauer 2004). Alternatively, many myxobolids and myxosporean species in general tend to cluster according to the family/order of the fish host, with this character being useful to distinguish genetically closely related species (e.g. Ferguson et al. 2008; Carriero et al. 2013; Vieira et al. 2017; Naldoni et al. 2018; Holzer et al. 2018; Liu et al. 2019). In the present study, our phylogenetic analysis showed that the three novel *Henneguya* species formed a well-supported sub-clade of perciform-infecting myxobolids, which included *Henneguya* sp. 3 PBS-2015 (KP030761), from gills of *Lates niloticus* from Lake Turkana, Kenya, and *Henneguya daoudi* (EU643625), from gills of another perciform fish, *Trichogaster trichopterus* from Malaysia (Fig. 5). The nearest sister clade included *Myxobolus neurophilus* (KF588564) and *Henneguya chaudhuryi* (KT279402), both of which infect perciform fish: *Perca flavescens* (yellow perch) and *Channa punctata* (spotted snakehead), respectively. The analysis thus revealed a stronger correlation between all three novel *Henneguya* spp. and their host, rather than the specific tissue in which they developed a pattern consistent across multiple groups of Myxobolidae as demonstrated previously (Liu et al. 2019).

Our taxonomic diagnosis incorporated consideration of whether either gill-infecting *Henneguya* species had already been described. Specifically, we considered their similarity with the only *Henneguya* known from gills (and mouth) of *L. calcarifer*: *H. latesi* Tripathi 1952, in barramundi from India. The description of that parasite is limited to host, tissue and morphometry (no DNA, no micrographs; Tripathi 1952). The taxon shares host and one of the two reported tissues (gills) with *H. setiuensis* n. sp. and *H. voronini* n. sp. but differs in most myxospore dimensions. We concluded that there was insufficient evidence to unambiguously identify either of our Malaysian gill-infecting species as *H. latesi*. Furthermore, given that we observed two genetically distinct *Henneguya* species within the gills, it is possible that the original description of *H. latesi* for “gills and mouth” could actually represent two distinct species. Determination of whether *H. latesi* represents a single taxon, distinct from any that we observed in Malaysian barramundi, would require redescription *H. latesi* from the type locality in India, with molecular and fine-scale tissue tropism observations.

Based on our limited sampling, we demonstrated the presence of at least three *Henneguya* species that parasitize barramundi in Malaysia. In our target population, these parasites occurred only at low prevalence and intensity, and without overt pathology. The health effects of any of these *Henneguya* species either singly or combined are unknown in populations of fish under conditions of stress or exposed to other health impacts from intense aquaculture, where *Henneguya*-related disease could emerge. Future work should concentrate on surveys from additional cultured fishes (both barramundi and grouper) from this region, under different stress levels and rearing conditions, to determine what other myxosporean species might be present and might be causing disease impacts.
